# Supplementary material for: Desmosomal cadherin association with Tctex-1 and cortactin-Arp2/3 drives perijunctional actin polymerization to promote keratinocyte delamination
Source: Nat Commun. 2018 Mar 13;9:1053. doi: 10.1038/s41467-018-03414-6 (PMC5849617; doi:10.1038/s41467-018-03414-6)
Supplement: Supplementary file 1 — Supplementary Information [file 41467_2018_3414_MOESM1_ESM.pdf]

Supplementary Information

Desmosomal cadherin association with Tctex-1 and cortactin-Arp2/3 drives perijunctional actin polymerization to promote keratinocyte delamination

Nekrasova et al.

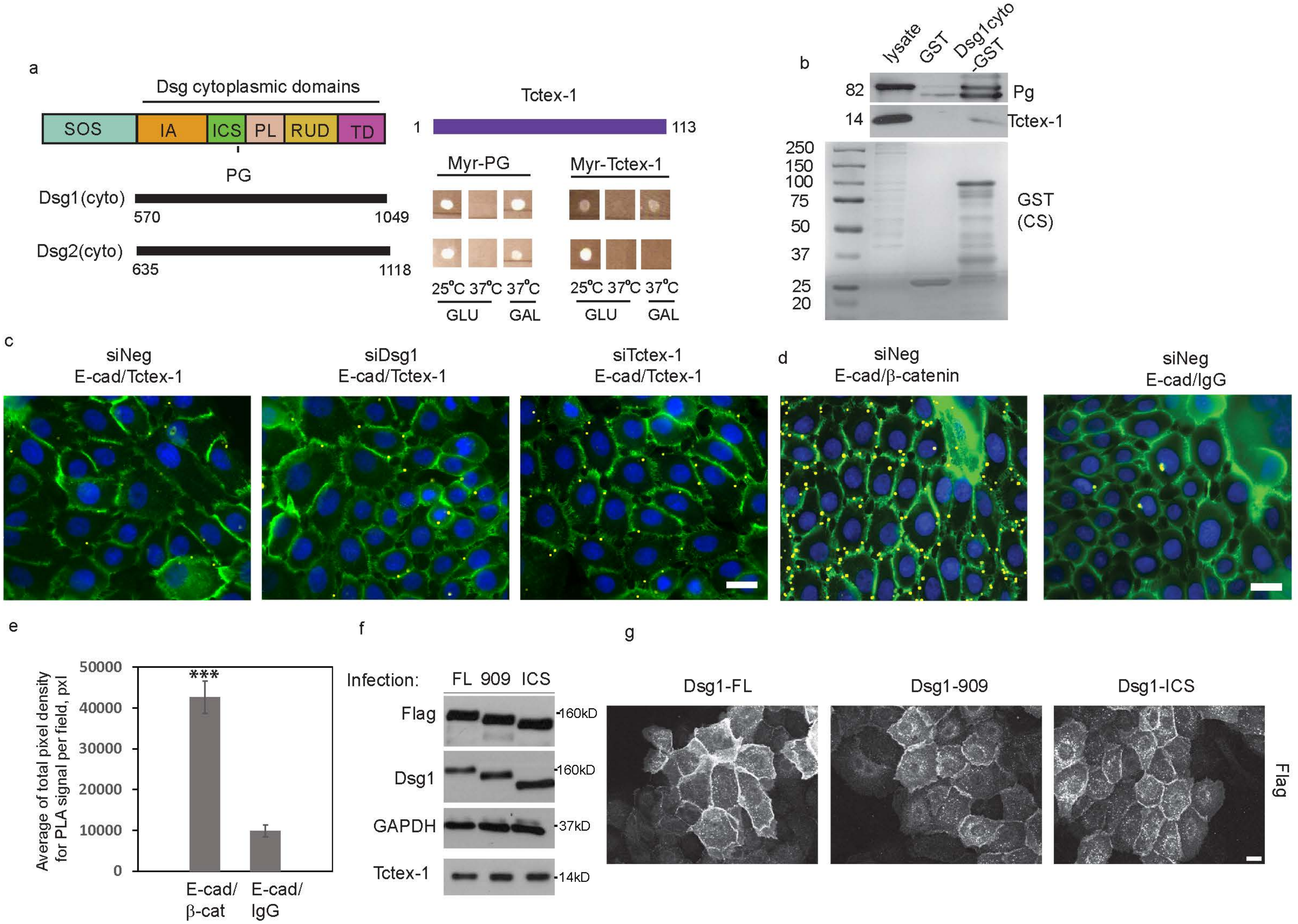

Supplementary Figure 1. Tctex-1 is a novel binding partner of Dsg1. (**a**) Yeast 2-hybrid (Y2H) analysis of interactions between Sos-tagged Dsg1 or Dsg2 cytoplasmic domains and Myr-Tctex-1 protein. Growth on galactose (GAL) at 37^{0}C indicates an interaction. Incubation on glucose (GLU) at 25^{0}C represents a permissive growth condition, while incubation at 37^{0}C on GLU is a control for temperature reversion. Interactions with Myr-PG serve as positive controls. IA-intracellular anchor domain; ICS, intracellular cadherin-like sequence domain; PL, intracellular proline-rich linker domain; RUD, repeat unit domain; DTD, desmoglein terminal domain. (**b**) Recombinant Dsg1 cytoplasmic tail-GST or GST were incubated with NHEK cell lysates. Retained proteins were immunoblotted for the presence of Tctex-1 and Pg (as a positive control for Dsg1 interactions). Expression of GST or Dsg1cyto-GST is shown in a Coomassie blue stained gel below the western blot (CS). (**c**) Proximity ligation assay (PLA) using primary antibodies directed against endogenous E-cad and Tctex-1 was performed on differentiated NHEKs with or without Dsg1 or Tctex-1 knockdown. PLA signal pseudocolored yellow; DAPI staining marks nuclei; Plakoglobin in green marks cell-cell borders. Close proximity was not observed for E-cad and Tctex-1. These representative control images correspond with the images and quantification included in Figure 1e, f and the western blot in Figure 1g. Scale bar = 20 µm (**d**) Proximity ligation assay (PLA) using primary antibodies directed against endogenous E-cad and b-catenin was performed on differentiated NHEKs. PLA signal pseudocolored yellow; DAPI staining marks nuclei; Plakoglobin in green marks cell-cell borders. Close proximity was observed for E-cad and b-catenin, as expected for this positive control. (**e**) PLA signal area was counted per field for each condition (>10 cells per field, 10 or more fields per condition) and averages were determined (representative experiment of three, ***$p<0.0001$, unpaired two-tailed t test). Scale bar = 20 µm (**f**) Western blot analysis of undifferentiated NHEKs retrovirally transduced with Dsg1-FL-Flag, Dsg1-909-Flag or Dsg1-ICS-Flag. Level of expression of ectopic constructs and endogenous Tctex-1 was analyzed using Flag, Dsg1 or Tctex-1 antibodies, respectively. GAPDH is a loading control. (**g**) Immunofluorescence staining of Flag in undifferentiated NHEKs retrovirally transduced with Dsg1-FL-Flag, Dsg1-909-Flag or Dsg1-ICS-Flag and switched to 1.2mM Ca^{2+} for 6h. Scale bar = 10 µm. Error bars represent standard error of the mean (SEM).

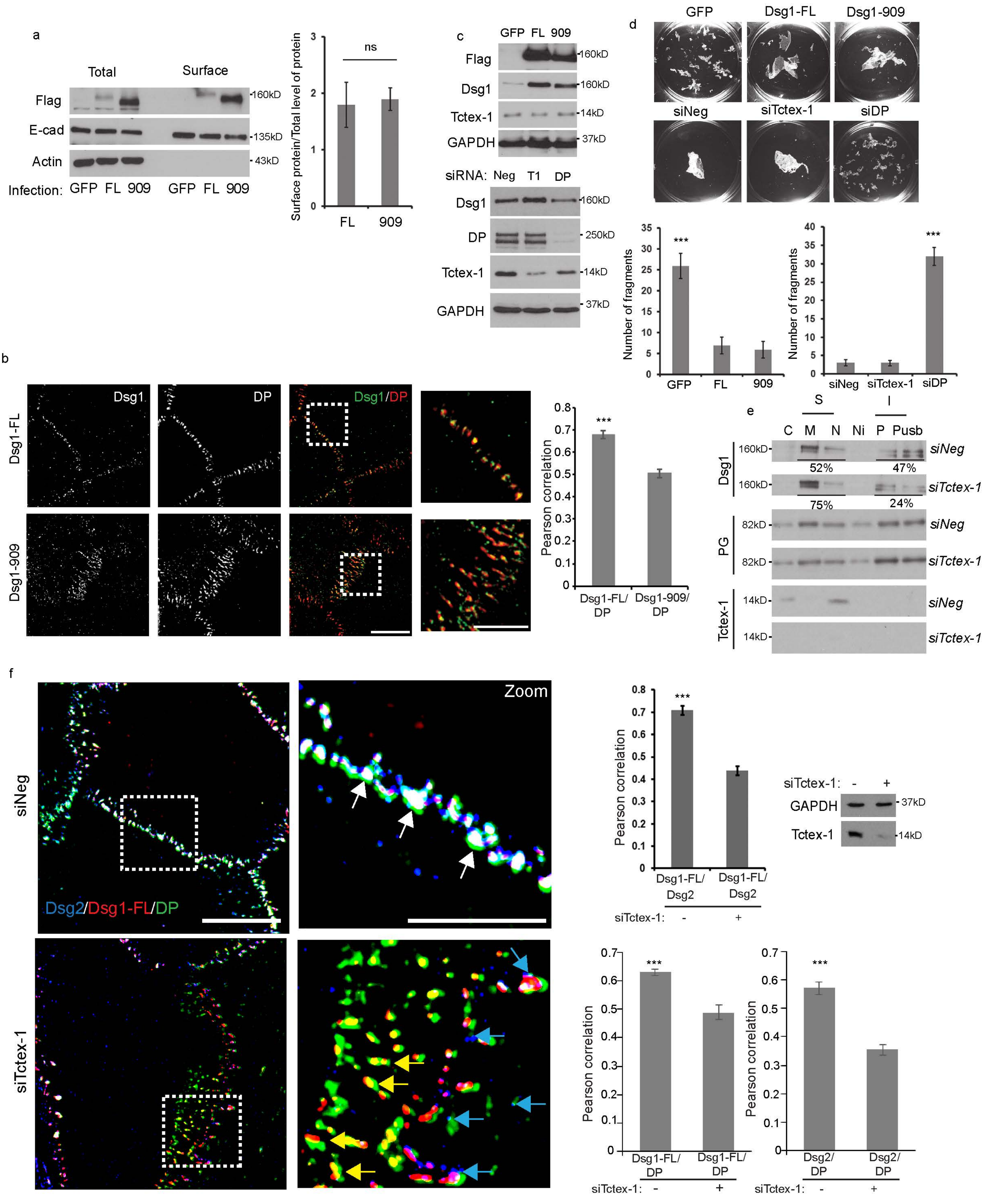

Supplementary Figure 2

Supplementary Figure 2. Tctex-1 is not required for Dsg1-positive desmosome assembly, but determines Dsg1's biochemical microenvironment. (**a**) Undifferentiated NHEKs retrovirally transduced with Dsg1-FL-Flag or Dsg1-909-Flag were cell surface biotinylated. Biotinylated proteins were isolated by streptavidin pull-down and levels of cell surface biotinylated, ectopic Dsg1 constructs and endogenous E-cadherin were identified by Western blot analysis. Actin is a loading control. Quantification of 4 independent experiments demonstrates no significant difference in cell surface expression for Dsg1-FL-Flag and Dsg1-909-Flag. (**b**) Structured illumination microscopy (SIM) images of ectopic Dsg1-FL-Flag or Dsg1-909-Flag in undifferentiated NHEKs. NHEKs were stained with antibodies recognizing Dsg1 (green) and endogenous desmoplakin (DP, red). Rectangles mark zoomed in areas, shown in the far right panels. Pearson's correlation coefficients for Dsg1-FL and DP, and Dsg1-909 and DP are shown in the graphs to the right of the images (data from one representative experiment out of 3 repeats, ***$p<0.0001$, unpaired two-tailed *t* test). (**c, d**) Confluent monolayers of undifferentiated NHEKs retrovirally transduced with GFP, Dsg1-FL-Flag, Dsg1-909-Flag or differentiated NHEKs treated with control, Tctex-1 or DP siRNA were subjected to a dispase mechanical dissociation assay in triplicate. Protein levels (expression or knockdown) are shown on the corresponding western blots (**c**), images of the monolayer fragmentation is presented (**d**) and quantifications of fragmentation under different conditions are shown on the bar graphs below the images (3 independent experiments per condition, $F=18.633$ (for ectopic protein expression) and $F=107.949$ (for knockdown conditions) ***$p<0.001$ one-way Anova with Tukey test). (**e**) Subcellular fractionation of differentiated NHEKs treated with control (siNeg) or Tctex-1 siRNA. Collected fractions were analyzed by western blotting for PG, Dsg1 and Tctex-1. C=cytoplasm, S=soluble membrane-bound fractions (M, membrane + N, nuclear), I=insoluble membrane bound fractions (P, pellet + Pusb, urea sample buffer solubilized pellet). Quantification of the percentage of Dsg1 in the soluble and insoluble membrane fractions are included. (**f**) SIM images of ectopic Dsg1-FL (red) co-stained with endogenous Dsg2 (blue) and DP (green) in undifferentiated NHEKs with or without Tctex-1 knockdown. White arrows indicate regions where Dsg1/Dsg2/DP localize together at a cell border. Yellow arrows indicate regions of Dsg1/DP co-localization, blue arrows mark regions of Dsg2/DP co-localization. Pearson's correlation coefficients for Dsg1-FL and Dsg2, Dsg1-FL and DP, and Dsg2 and DP, with or without Tctex-1 knockdown are shown in the graphs to the right of the images (data from one representative experiment out of 3 repeats, ***$p<0.0001$ for Dsg1-FL/Dsg2, $p<0.0003$ for Dsg1/DP and $p<0.0001$ for Dsg2/DP, unpaired two-tailed *t* test). The level of Tctex-1 knockdown is shown on the corresponding Western blot. Scale bars = 10 µm. Error bars represent SEM.

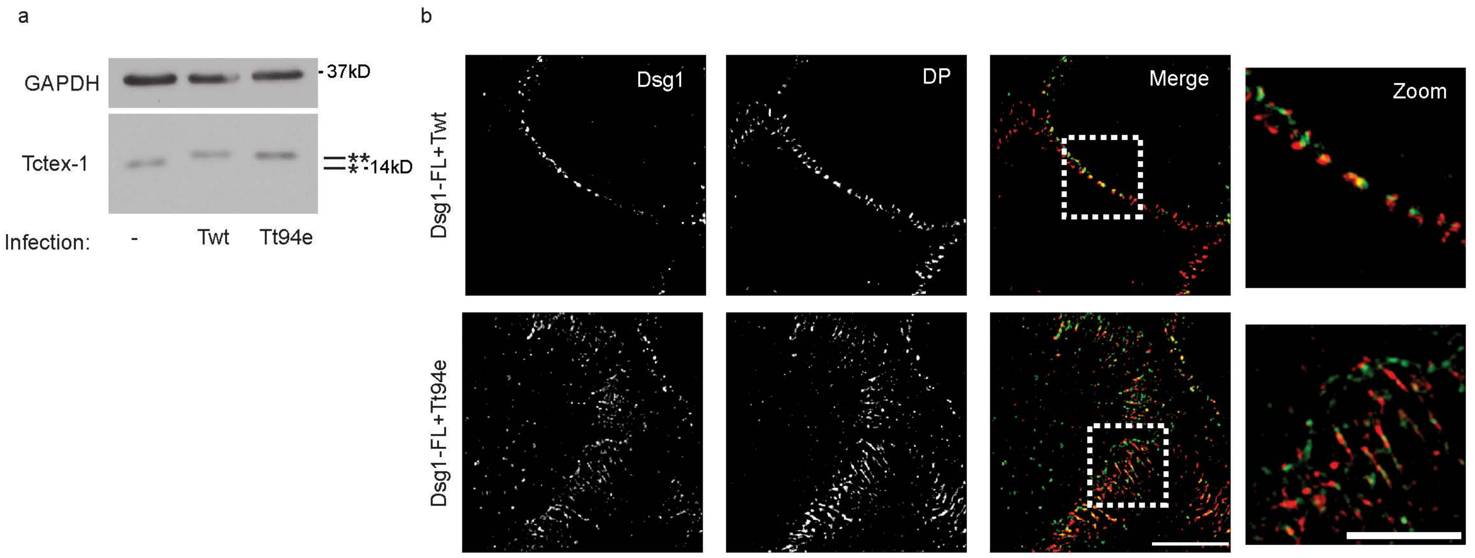

Supplementary Figure 3. Uncoupling Tctex-1 from the dynein complex affects Dsg1 accumulation at the insoluble plasma membrane pool. (**a**) Western blot analysis of expression levels for endogenous Tctex-1 and ectopically expressed Tctex-1 constructs: Tctex-1-wt-Flag (Twt) and Tctex-1-t94e-Flag (Tt94e), in NHEKs. Note that the Flag tag increases the molecular weight of ectopic Tctex-1 proteins, resulting in a slight shift on the Western blot, and marked (**), while (*) marks the molecular weight of endogenous Tctex-1. GAPDH serves as a loading control. (**b**) Structured illumination microscopy images of undifferentiated NHEKs retrovirally transduced with Dsg1-FL-Flag and Tctex-1-wt-Flag (upper panels) or Dsg1-FL-Flag and Tctex-1-t94e-Flag (lower panels). NHEKs were stained with anti-Dsg1 antibody (green) and anti-DP (red). Rectangles mark zoomed in areas. Scale bars = 10 μm.

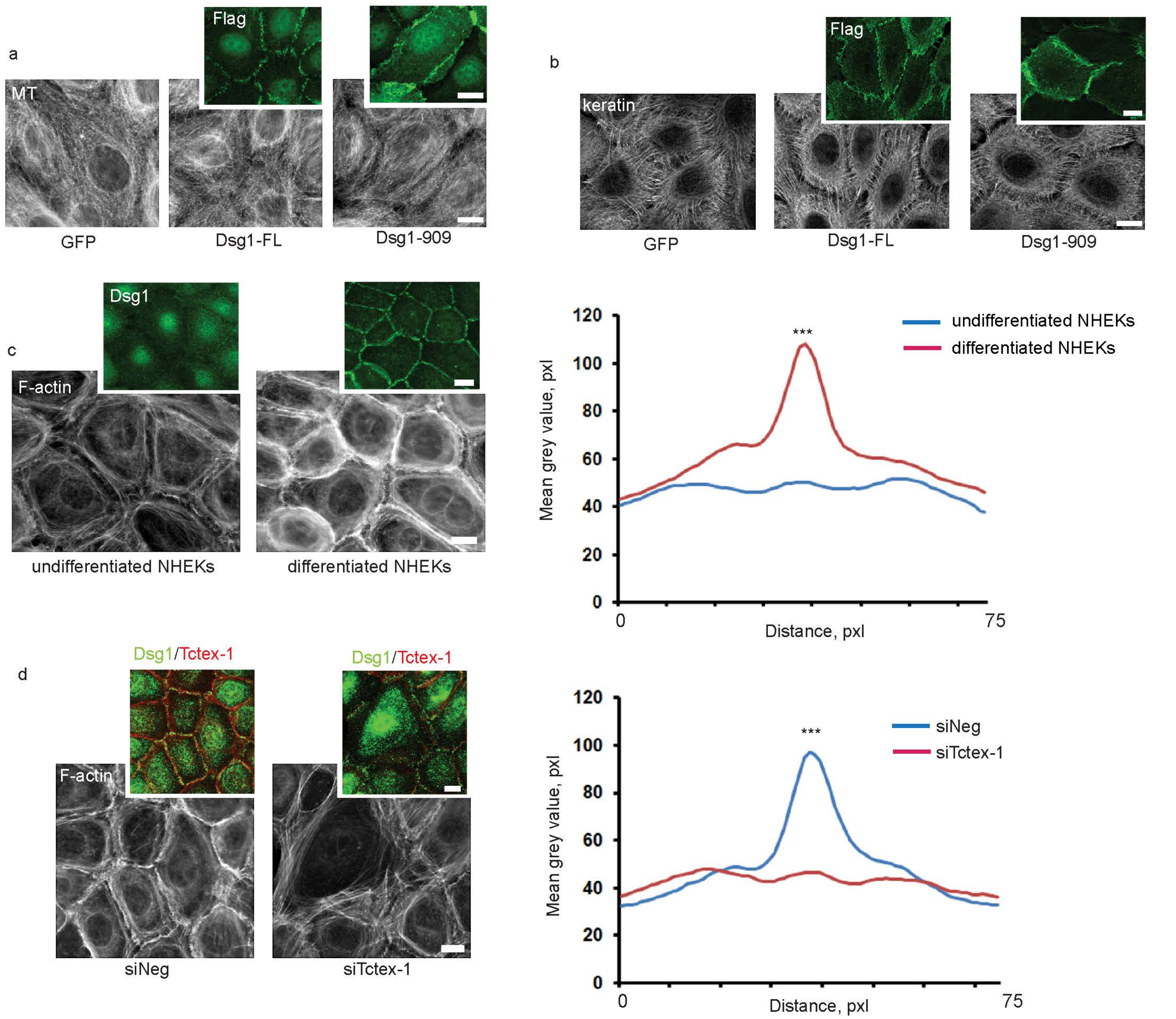

Supplementary Figure 4. Dsg1-Tctex-1 interactions do not affect microtubule or intermediate filament network organization but are involved in actin reorganization at cell-cell junctions during keratinocyte differentiation. (**a**) Immunofluorescence staining of microtubules (MT) and Flag (top insets, where applicable) in undifferentiated NHEKs retrovirally transduced with GFP, Dsg1-FL-Flag or Dsg1-909-Flag. (**b**) Immunofluorescence staining for keratin 5 /14 and Flag (shown in top inserts where applicable) in undifferentiated NHEKs expressing GFP, Dsg1-FL-Flag or Dsg1-909-Flag. (**c**) Immunofluorescence staining for Dsg1 (green) and F-actin labeled with fluorescently conjugated phalloidin in undifferentiated and differentiated NHEKs. Line scan analyses of cortical F-actin intensities at the cell-cell interface in undifferentiated and differentiated NHEKs are presented to the right of representative images (3 independent experiments, at least 50 borders analyzed per condition in each experiment, ***$p<0.001$, unpaired two-tailed t test). (**d**) NHEKs treated with control or Tctex-1 siRNA were differentiated for 3 days and stained for F-actin, Dsg1 (green) and Tctex-1(red). Line scan analyses of cortical F-actin intensities at the cell-cell interface in control or Tctex-1 silenced, differentiated NHEKs are shown on the right (3 independent experiments, at least 50 borders were analyzed per condition in each experiment, ***$p<0.001$, unpaired two-tailed t test). Scale bars = 10 μm.

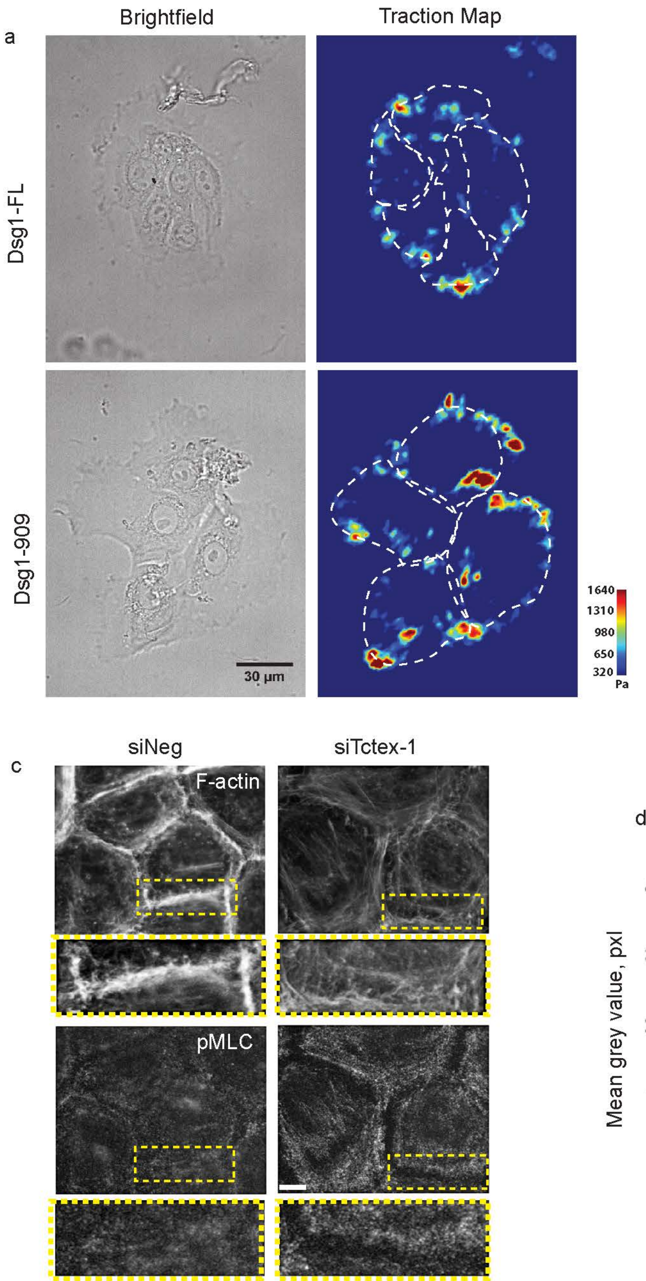

Supplementary Figure 5. Dsg1-Tctex-1 interactions decrease cells contractility. (**a**) To measure cell-ECM traction forces NHEKs expressing Dsg1-FL or Dsg1-909 were plated on 4.1 kPa polyacrylamide gels coated with collagen. Images of 40nm fluorescent beads embedded in the gel and brightfield images of the colonies were taken before and after removal of the colony via SDS. Bead displacement between the images taken before and after SDS treatment was used to calculate traction forces. Distribution of strain energy density per cell colony is shown on the heatmaps. Scale bar = 30 µm. (**b**) Quantification of the strain energy per area for NHEKs with Dsg1-FL or Dsg1-909. To evaluate colony contractility, the energy required to produce the observed gel deformations (strain energy) was normalized against colony area to account for the linear relation between cell area and strain energy. 10 colonies were analyzed per condition (Box covers 25-75th percentile, the mean is indicated by an open square and the median is indicated by the center line). Data from one representative experiment are shown (2 independent experiments, ***p<0.001, unpaired two-tailed t test). (**c**) NHEKs treated with control or Tctex-1 siRNA were differentiated for 3 days and stained for F-actin and phosphorylated myosin light chain (pMLC). Scale bar = 10 µm (**d**) Quantification of pMLC intensity at the junctional area in differentiated NHEKs with or without Tctex-1 knockdown. Box boundaries indicate the 25-75th percentile of measured intensity in pixels, middle bars depict the mean of compiled data sets, whiskers represent the maximum and the minimum of the measured intensities (at least 80 borders were analyzed per condition, 2 independent experiments, ***p<0.001, unpaired two-tailed t test.). Error bars represent SEM.

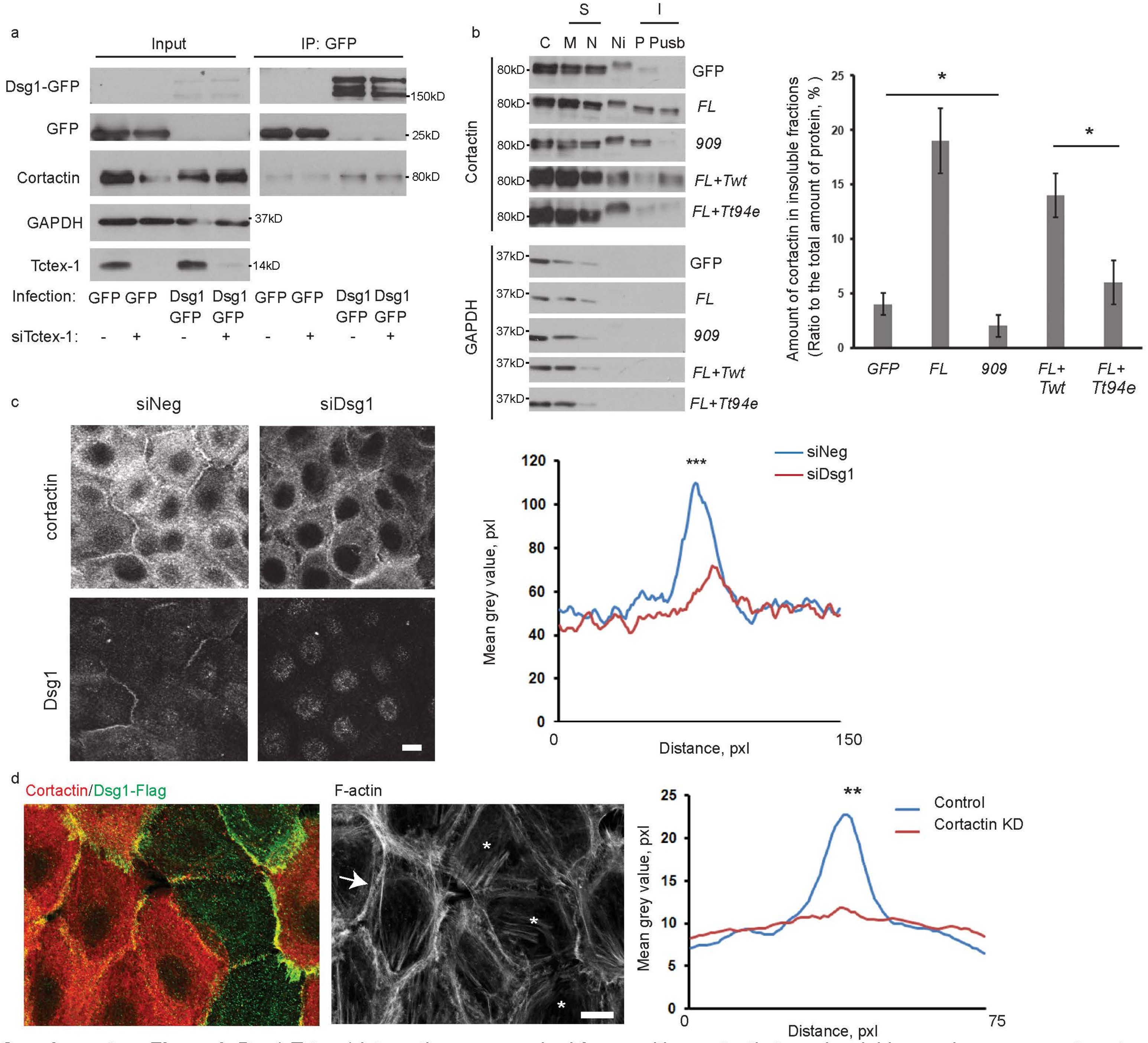

Supplementary Figure 6. Dsg1-Tctex-1 interactions are required for recruiting cortactin to an insoluble membrane compartment to promote perijunctional actin reorganization. (**a**) Western blot of GFP immunoprecipitates from undifferentiated NHEKs expressing GFP or Dsg1-GFP, with or without Tctex-1 knockdown, probed for Dsg1, GFP, cortactin and Tctex-1. GAPDH serves as a loading control. (**b**) Subcellular fractionation of undifferentiated NHEKs retrovirally transduced with: GFP, Dsg1-FL-Flag (FL), Dsg1-909-Flag (909), Dsg1-FL-Flag and Tctex-1-wt-Flag (FL+Twt) or Dsg1-FL-Flag and Tctex-1-t94e-Flag (FL+Tt94e). Collected fractions were analyzed for cortactin and GAPDH was included as a loading control. C=cytoplasm, S=soluble membrane-bound fractions (M, membrane + N, nuclear), I=insoluble membrane bound fractions (P, pellet + Pusb, urea sample buffer solubilized pellet). The percentage of cortactin in insoluble membrane fractions for all conditions is quantified to the right (3 independent experiments; F=6.767, *$p<0.05$ one-way Anova with Tukey test; *$p<0.05$ unpaired two-tailed t test). (**c**) Immunofluorescence staining for cortactin and Dsg1 in differentiated NHEKs with or without Dsg1 knockdown. Line scan analyses of cortactin intensity at the cell-cell interfaces in differentiated NHEKs with or without Dsg1 expression (at least 25 borders from Dsg1 expressing and 25 borders from Dsg1-silenced cells were analyzed ***$p<0.001$, unpaired two-tailed t test). (**d**) Undifferentiated NHEKs retrovirally transduced with Dsg1-FL-Flag were treated with cortactin siRNA and stained for cortactin (red), Dsg1 (green) and F-actin utilizing fluorescently conjugated phalloidin. Asterisks mark cortactin silenced cells as assessed by lack of cortactin staining (see left panel). The arrow indicates concentrated fluorescence staining of perijunctional F-actin in NHEKs expressing Dsg1-FL and endogenous cortactin. Line scan analyses of cortical F-actin intensities at the cell-cell interface in undifferentiated NHEKs expressing Dsg1-FL-Flag with or without cortactin knockdown is shown to the right (at least 25 borders from cortactin expressing and 25 borders from cortactin-silenced cells were analyzed from the same fields, **$p<0.01$, unpaired two-tailed t test).
Scale bar = 10 μm. Error bars represent SEM.

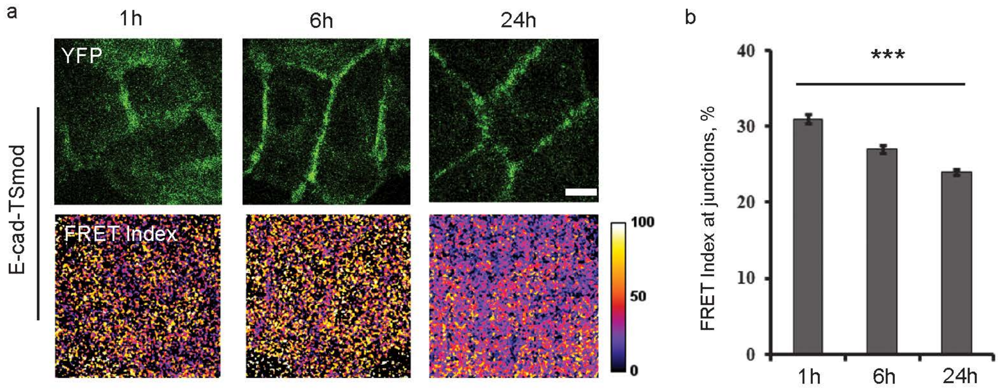

Supplementary Figure 7. Tension on E-cadherin increases during a Ca^{2+} switch time course. (**a**) An E-cadherin FRET tension sensor was expressed in undifferentiated NHEKs. NHEKs were incubated in 1.2mM Ca^{2+} for 1h, 6h or 24h before measuring the FRET index. Fluorescence images for the YFP channel (indicating E-cad-TSmod expression, green) and corresponding maps of the FRET index (bottom panels) are shown for each time point. Scale bar = 10 μm. (**b**) FRET index for E-cad-TSmod was measured at cell-cell contacts in undifferentiated NHEKs (3 independent experiments; F=45.564, ***p<0.001 one-way Anova with Tukey test). Error bar represents SEM.

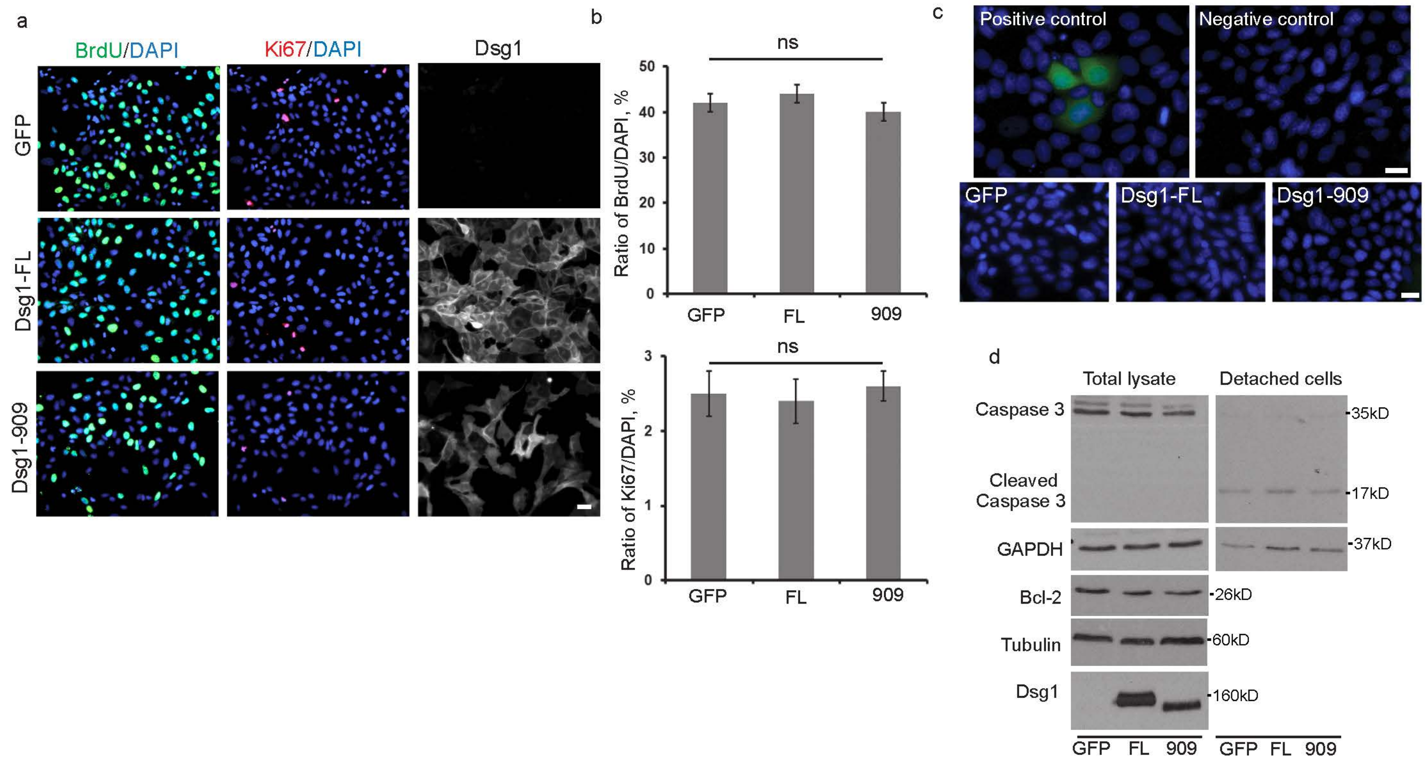

Supplementary Figure 8. Expression of ectopic Dsg1 proteins in MDCK cells does not stimulate proliferation or apoptosis. (**a**) Immunofluorescence staining for BrdU (green), Ki67 (red) and Dsg1 (grey) in MDCK cells expressing GFP, Dsg1-FL-Flag or Dsg1-909-Flag. DAPI marks nuclei. (**b**) Quantification of BrdU and Ki67 positive cells to the total amount of cells in the field (3 independent experiment; ns, non-significant, F=1.184 (for BrdU) and F=0.072 (for Ki67) one-way Anova). (**c**) Representative fluorescent images for TUNEL (DNA strand breaks) in MDCK cells expressing GFP, Dsg1-FL or Dsg1-909 mutant. Positive control: MDCK cells were incubated with micrococcal nuclease to induce DNA strand breaks before labeling; negative control: MDCK cells were labeled without terminal transferase to depict the background staining. TUNEL assay yielded no positive staining in MDCK cells expressing GFP, Dsg1-FL or Dsg1-909. DAPI marks nuclei. (**d**) Western blot analysis of expression levels for endogenous Caspase 3 (total and cleaved proteins) in total cell lysate and lysates made from detached cells harvested from the media; endogenous Bcl-2 in total cell lysate, and ectopic Dsg1 proteins in total cell lysate. GAPDH and Tubulin are loading controls. Scale bars = 10 μm. Error bars represent SEM.

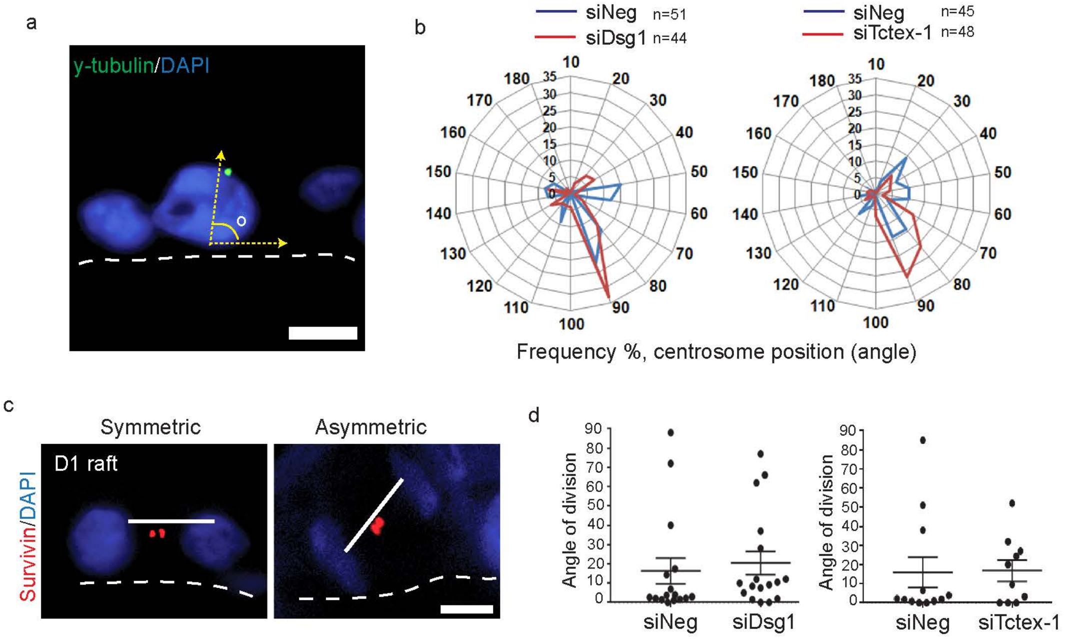

Supplementary Figure 9. Dsg1-Tctex-1 interactions are not required for symmetric/asymmetric divisions in NHEKs during stratification. (**a**) Representative fluorescent image of y-tubulin (centrosome) in a basal cell of a Day 1 raft. DAPI marks nuclei. The position of y-tubulin relative to the nucleus and the basal membrane was measured as an angle. (**b**) Quantification of the centrosome angle positioning in control (siNeg), Dsg1 or Tctex-1 knockdown conditions (3 independent experiments, non-significant difference between control and knockdown conditions, unpaired two-tailed t test, $p=0.4$ for siNeg/siDsg1 and $p=0.2$ for siNeg/siTctex-1). (**c**) Representative fluorescence images of survivin staining in telophase cells in D1 rafts. DAPI marks nuclei. (**d**) Quantification of basal cell division angles in D1 rafts in control (siNeg), Dsg1 or Tctex-1 knockdown conditions (3 independent experiments per condition, dots are showing individual measurements). Scale bars are 10 μm. Error bars represent SEM.

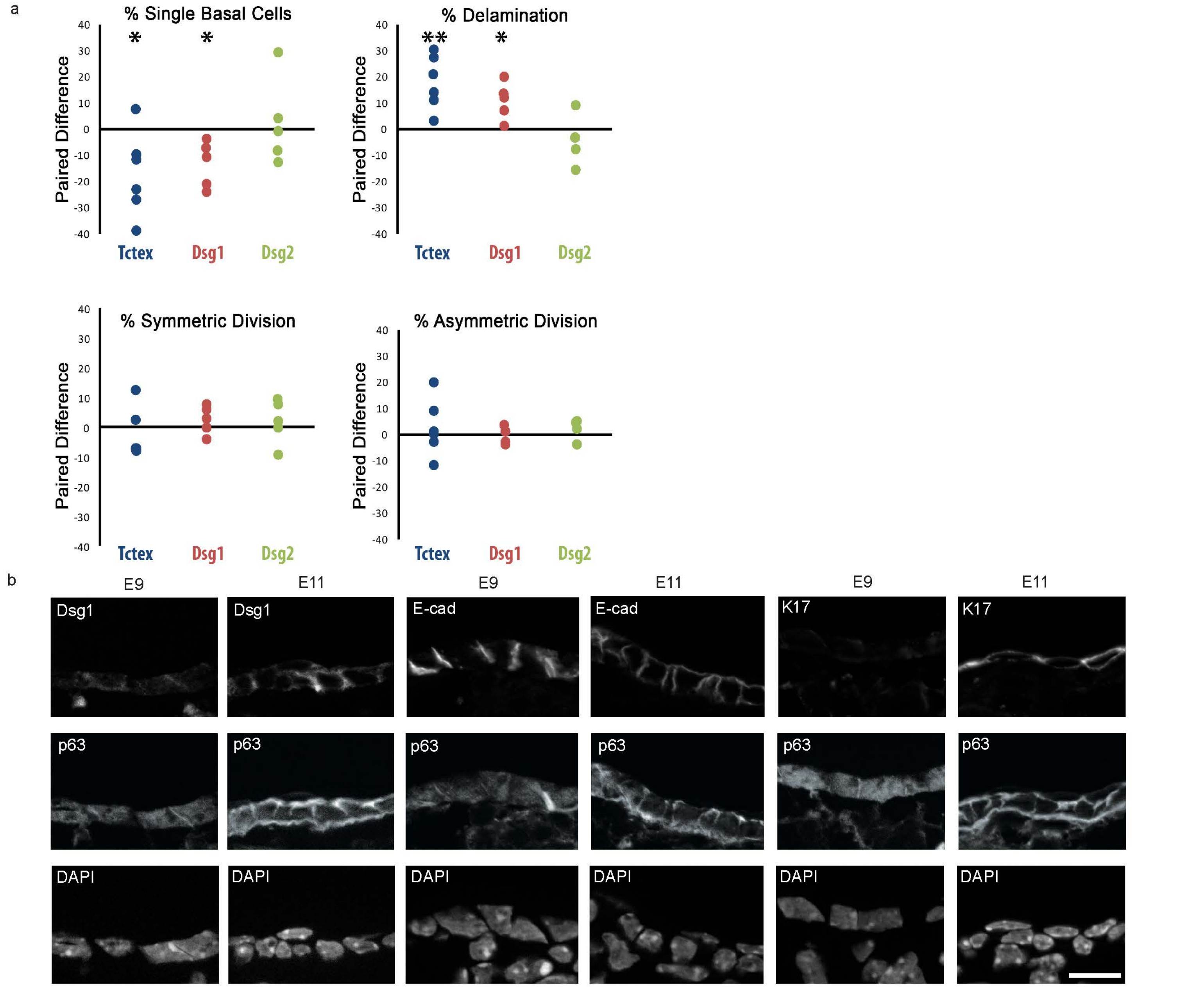

Supplementary Figure 10. Dsg1-Tctex-1 interactions promote basal cell delamination. (**a**) Paired differences were calculated for control (siNeg) and treatment (siTctex-1, siDsg1 or siDsg2) conditions for the following percentages: single basal cells events, delamination events, symmetric division events and asymmetric division events observed in D1 rafts. Each point on the scatter plot represents a paired difference comparing the control and a treatment condition. 6 independent experiments were performed for siNeg and siTctex-1 conditions, 5 independent experiments were performed for siNeg and siDsg1conditions and 5 independent experiments were performed for siNeg and siDsg2 conditions. Each independent experiment used the same NHEK clone for the control and treatment condition, but different clones were used for each experiment. A positive difference represents a decrease in the treatment group compared with control, whereas a negative difference represents an increase. Control and knockdown conditions were compared using a paired two-tailed t test. Tctex-1 knockdown significantly increased the percentage of single basal cells events observed in population with *p=0.0446 and 95% confidence interval (CI): 0.6125 to 34.05; Dsg1 knockdown significantly increased the percentage of single basal cells events observed in population with *p=0.0238 and 95% CI: 2.969 to 24.23; Dsg2 knockdown does not affect the percentage of single basal cells events (p=0.7997 with 95% CI: -22.48 to 18.48). Tctex-1 knockdown significantly decreased the percentage of delamination events with **p=0.0083 and 95%CI: -28.29 to -6.879; Dsg1 knockdown significantly decreased the percentage of delamination events with *p=0.0288 and 95%CI: -19.41 to -1.794; Dsg2 knockdown does not affect the percentage of delamination events (p=0.3189 with 95%CI: -6.63 to 15.83. None of the knockdowns affected the percentage of symmetric or asymmetric division events observed in the population. The bar graphs of population analyses that correspond to these scatter plots are shown in Figure 8d, e and f. (**b**) Expression of Dsg1, E-cadherin, Keratin 17 and p63 in developing epidermis. Immunofluorescence images of murine epidermis at E9 or E11. Tissues were stained for Dsg1, E-cadherin (E-cad), Keratin 17 (K17) and p63. p63 staining marks ectoderm. K17 marks periderm. DAPI staining marks nuclei. Scale bar = 20 μm.

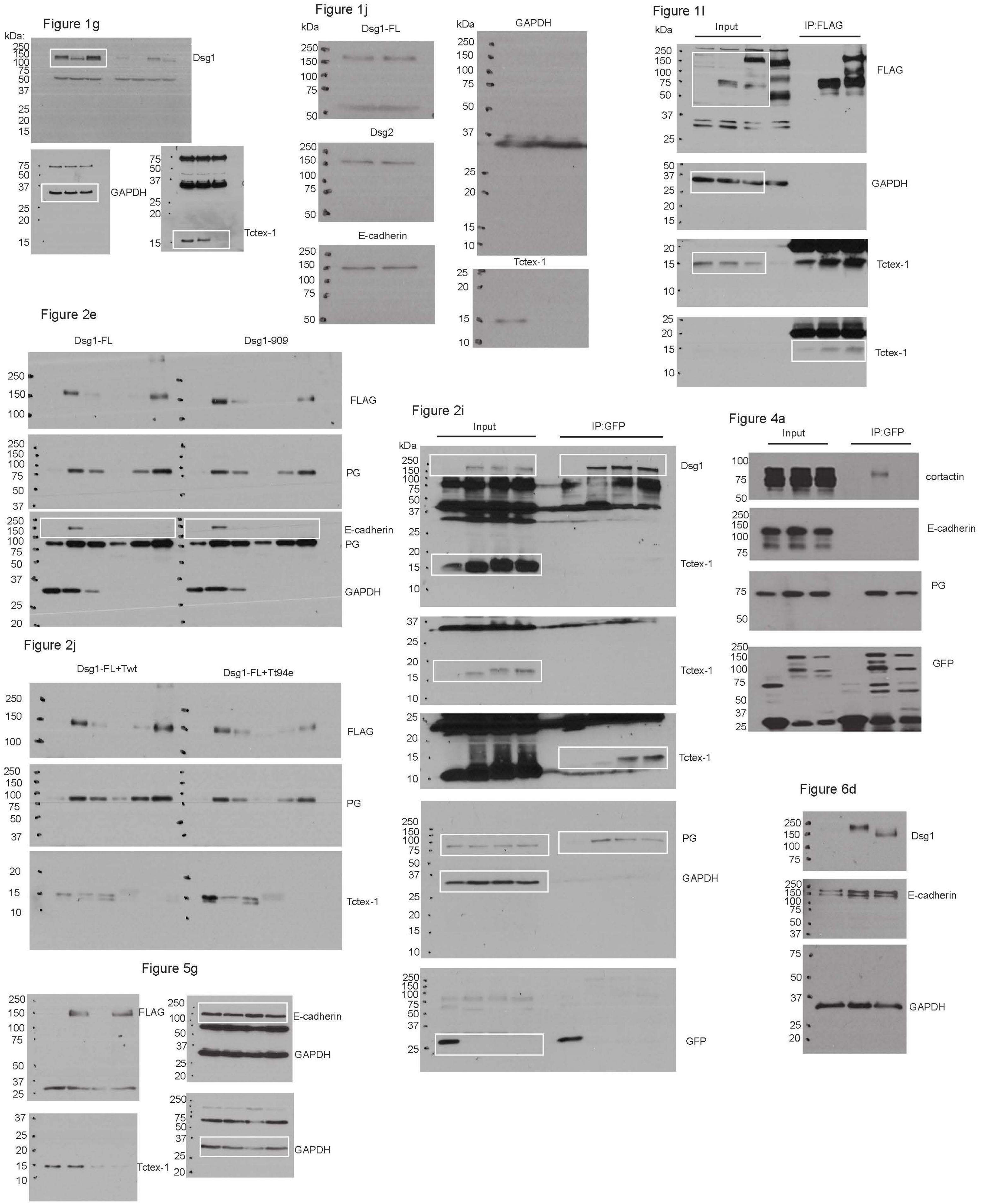

Supplementary Figure 11. Uncropped Western blots related to Figures 1g, 1j, 1l, 2e, 2i, 2j , 4a, 5g, 6d.

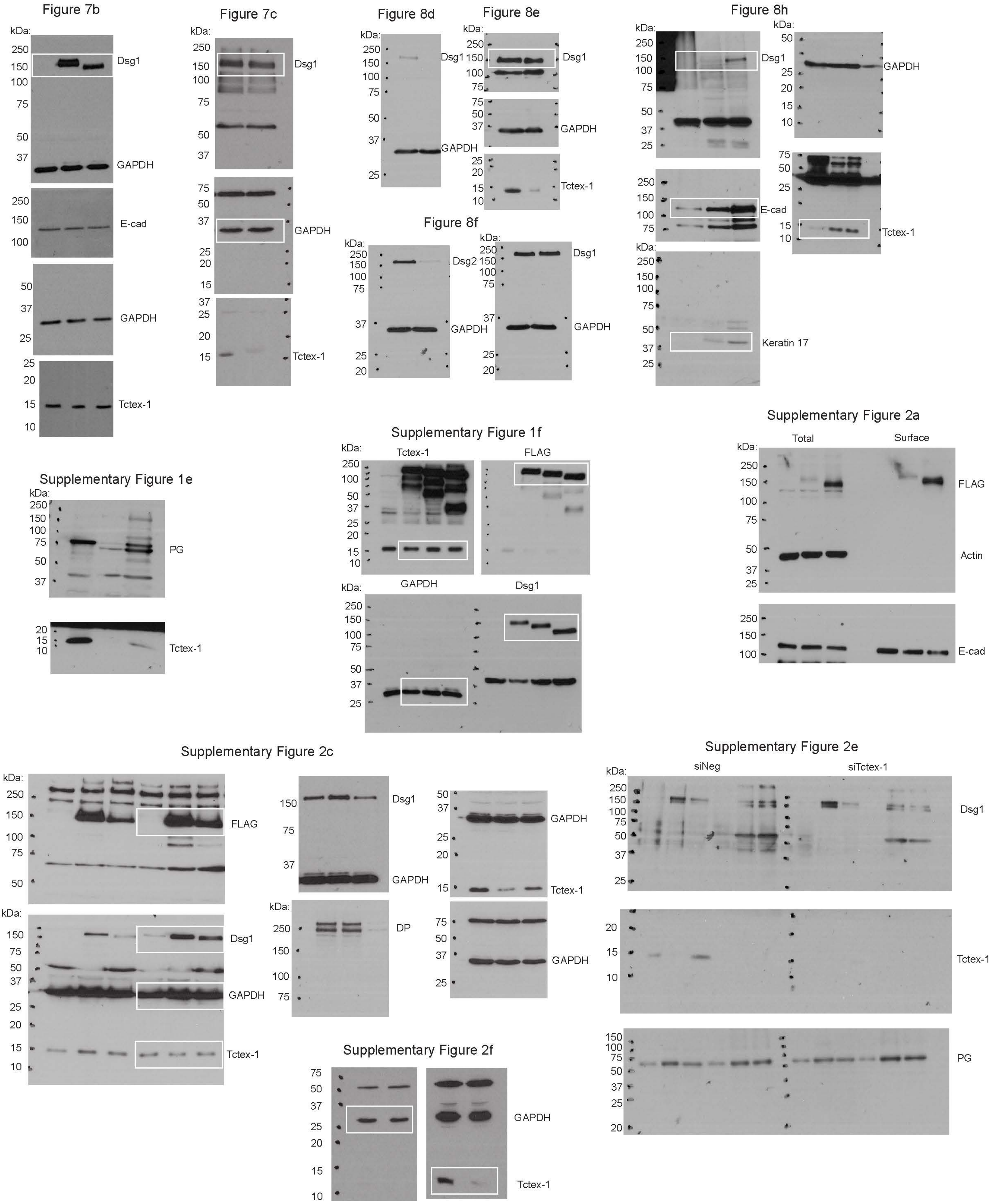

Supplementary Figure 12. Uncropped Western blots related to Figures 7b, 7c, 8d, 8e, 8f, 8h and Supplementary Figures 1e, 1f and 2a, 2c, 2e, 2f.

Supplementary Figure 3a

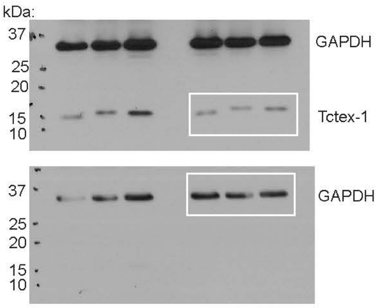

Supplementary Figure 6a

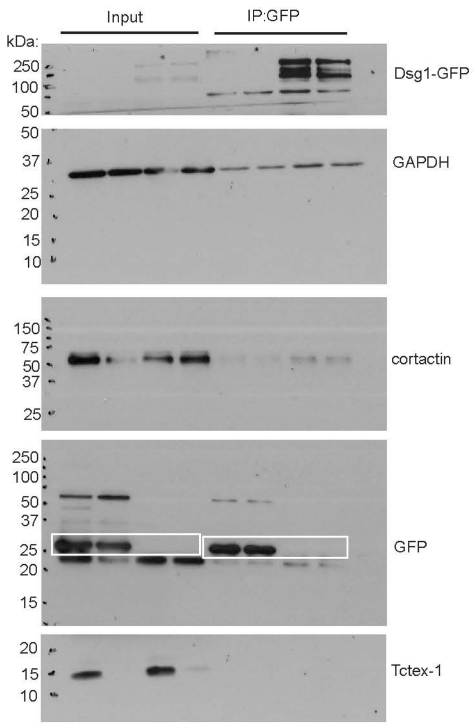

Supplementary Figure 8d

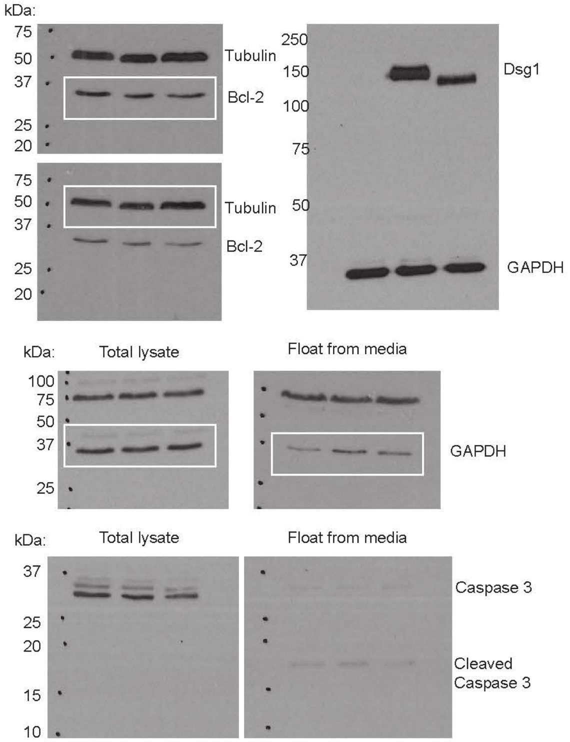

Supplementary Figure 6b

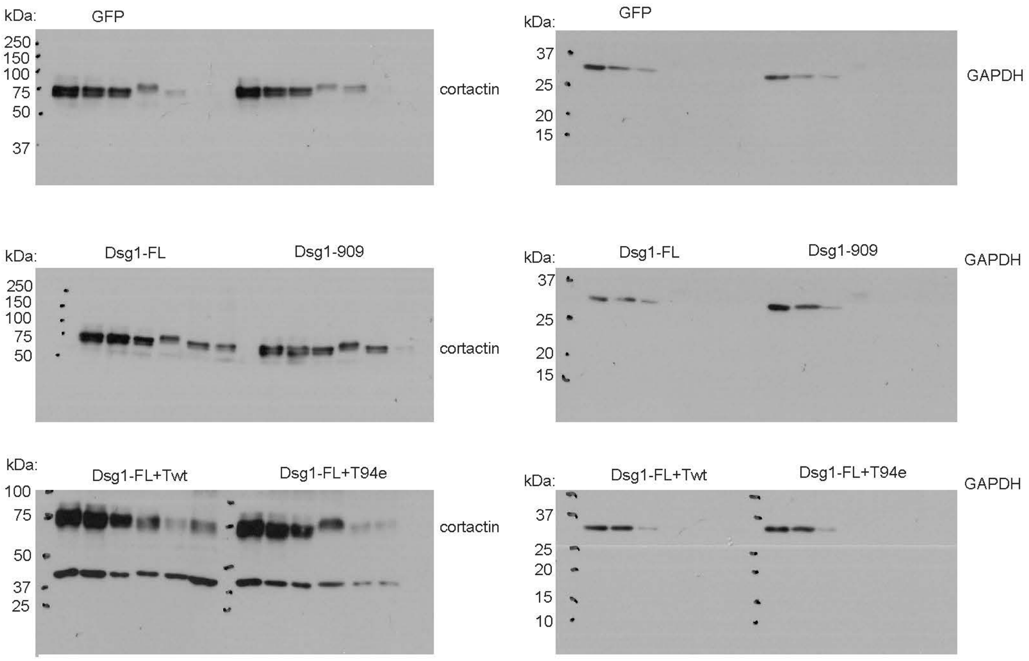

Supplementary Figure 13. Uncropped Western blots related to Supplementary Figures 3a, 6a, 6b, 8d.
