# Supplementary material for: Desmosomal cadherin association with Tctex-1 and cortactin-Arp2/3 drives perijunctional actin polymerization to promote keratinocyte delamination
Source: Nat Commun. 2018 Mar 13;9:1053. doi: 10.1038/s41467-018-03414-6 (PMC5849617; doi:10.1038/s41467-018-03414-6)
Supplement: Supplementary file 3 — Description of Additional Supplementary Files [file 41467_2018_3414_MOESM3_ESM.pdf]

Description of Additional Supplementary Files

File Name: Supplementary Movie 1
Description: E-cadherin-YFP-labeled cell-cell border of NHEKs expressing mCherry before and after laser ablation.

File Name: Supplementary Movie 2
Description: E-cadherin-YFP-labeled cell-cell border of NHEKs expressing Dsg1-FL before and after laser ablation.

File Name: Supplementary Movie 3
Description: E-cadherin-YFP-labeled cell-cell border of NHEKs expressing Dsg1-909 before and after laser ablation.

File Name: Supplementary Movie 4
Description: Behaviour of MDCK cells expressing GFP (green); >12 hours of recording.

File Name: Supplementary Movie 5
Description: Behavior of MDCK cells expressing Dsg1-GFP (green); >12 hours of recording.
